# Hepatotoxicity associated with anti-neoplastic agents: a pharmacovigilance analysis of the Food and Drug Administration Adverse Event Reporting System database

**DOI:** 10.3389/fimmu.2025.1665425

**Published:** 2025-12-19

**Authors:** Lei Shi, Yan Wang, Ying Qu, Rong Chen

**Affiliations:** 1 Department of Pharmacy, The Third Affiliated Hospital of Soochow University/The First People’s Hospital of Changzhou, Changzhou, China; 2 Department of Pharmacy, Kangda College of Nanjing Medical University, Lianyungang, China

**Keywords:** anti-neoplastic agents, disproportionality analysis, FAERS, hepatotoxicity, pharmacovigilance

## Abstract

**Introduction:**

Hepatotoxicity is commonly observed in patients undergoing chemotherapy. However, the clinical features and outcomes of hepatotoxicity associated with anti-neoplastic agents remain unclear. In this study, we investigated the characteristics and risk factors of hepatotoxicity associatedwith anti-neoplastic agents.

**Methods:**

We conducted a retrospective pharmacovigilance analysis using data acquired from the FDA Adverse Event Reporting System (FAERS) database (Q1–2004 to Q3 2024). Hepatotoxicity risk was assessed by disproportionality analysis, while LASSO and multivariate logistic regression were applied to control for potential confounders. Finally, we analyzed the time duration to the onset of hepatotoxicity.

**Results:**

A total of 56 anti-neoplastic agents exhibited positive signals for hepatotoxicity, involving 4,195 reports. Female patients (46.50%) were more frequently affected than males (26.70%), with a median age of 56 years. 627 patients (14.95%) experienced fatal or life-threatening outcomes. The top three drugs with the highest Reporting Odds Ratio (ROR) values were mercaptopurine (ROR = 26.57), pegaspargase (ROR = 13.67) and blinatumomab (ROR = 11.93). Most events occurred within the first month (44.18%) and the median TTO value in the Fatal group (22.5 days) was shorter than that in the non-fatal group (42 days) (p < 0.05). Furthermore, Weibull shape parameter (WSP) analysis indicated that 20 of the top 30 drugs were random failure models.

**Discussion:**

This analysis profiles hepatotoxicity signals for anti-neoplastic agents but reveals a major methodological gap: without using a validated causality tool like the updated RUCAM, FAERS data cannot confirm druginduced liver injury (DILI). Future studies should integrate RUCAM to improvespecificity and clinical relevance.

## Graphical Abstract

**Figure:**
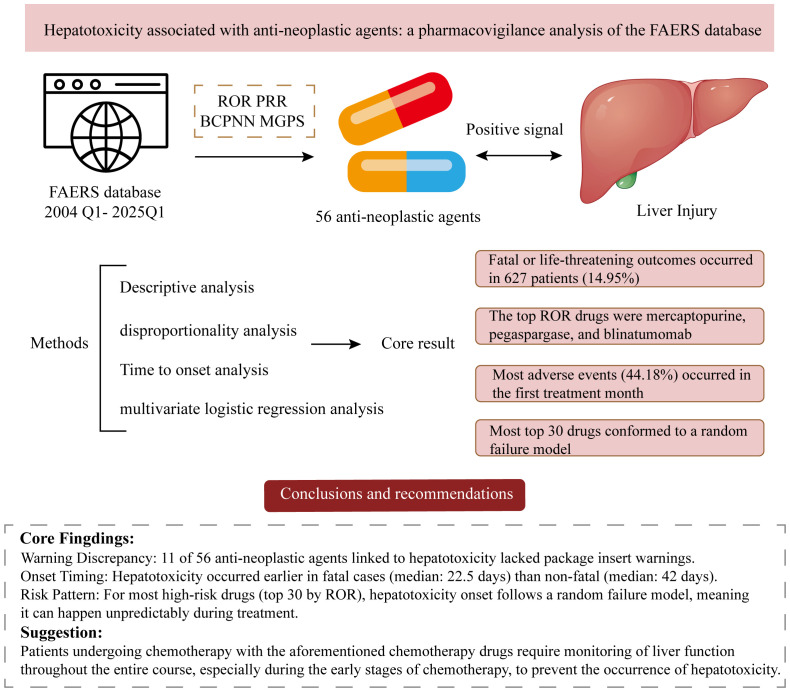

## 1 Introduction

According to 2019 estimates from the World Health Organization (WHO), cancer was the first or second leading cause of death prior to the age of 70 years in 112 out of 183 countries (1). Currently, chemotherapy remains one of the cornerstone modalities for the treatment of malignant tumors, playing a vital role in inhibiting tumor progression and prolonging patient survival (2, 3). The liver, as the central organ for drug metabolism in humans, plays a critical role in the metabolic processing of anti-neoplastic agents. However, this physiological function inevitably induces hepatotoxicity during the administration of anti-neoplastic agents (4); this is commonly known as drug-induced liver injury (DILI) (5, 6). DILI not only diminishes therapeutic efficacy but may also progress to acute liver failure, posing severe threats to patient survival and quality-of-life (7).

From a pathophysiological perspective, DILI is conventionally categorized into three phenotypic patterns based on the relative elevations of specific serum biomarkers. This classification is typically determined using the R value, a diagnostic ratio calculated as R = (Alanine Aminotransferase [ALT]/Upper Limit of Normal [ULN])/(Alkaline Phosphatase [ALP]/ULN). The R value defines the specific phenotypic pattern, as follows: [I] hepatocellular injury, characterized by the predominant elevation of ALT and defined by an R value ≥ 5; [II] cholestatic injury, characterized by the predominant elevation of ALP, often accompanied by levels of elevated γ-glutamyl transferase (GGT), and defined by an R value ≤ 2; and [III] mixed liver injury, which exhibits features of both I and II and is defined by an R value between 2 and 5 (8). The current laboratory diagnosis of DILI primarily relies on the dynamic monitoring of serum biochemical markers, including ALT, aspartate aminotransferase (AST), GGT, ALP, and total bilirubin (TBIL) levels (9). However, because such biomarkers are nonspecific, the definitive diagnosis of DILI still relies on the Roussel Uclaf Causality Assessment Method (RUCAM) as the gold standard (10, 11). Given the severity of hepatotoxicity associated with chemotherapy, establishing systematic protocols to evaluate liver function and identifying the potential hepatotoxic risks of anti-neoplastic agents have become crucial clinical imperatives. Consequently, the comprehensive analysis of hepatotoxicity associated with anti-neoplastic agents is of significant importance for optimizing the therapeutic management of oncology.

With the increasing utilization of anti-neoplastic agents, research relating to the hepatotoxic effects of these agents has garnered substantial research attention. For instance, Lai et al. (12) conducted a retrospective study investigating the clinical characteristics, causative agents and outcomes of DILI in Chinese pediatric populations. These authors found that adolescents exhibited a higher prevalence of moderate-to-severe DILI and greater risks of critical hepatic impairment than younger children; the primary causative agents were anti-neoplastic agents (25.9%), antibiotics (21.5%), and traditional Chinese medicines (13.7%). Another retrospective analysis of hepatotoxicity-associated adverse drug reactions in China, carried out between 2012 and 2016, demonstrated a consistent upwards trend in reported cases, particularly among males and elderly populations, with the group featuring patients over 80 years-of-age exhibiting a significantly higher incidence of DILI than the general population (13). Nevertheless, previous studies predominantly focused on single dimensions with limited sample sizes and failed to comprehensively elucidate the association between anti-neoplastic agents and hepatotoxicity. Therefore, multi-dimensional evaluation of this relationship through large-scale databases carries paramount scientific significance.

The U.S. Food and Drug Administration Adverse Event Reporting System (FAERS), a publicly accessible database featuring adverse event reports and medication error submissions, has been extensively utilized for pharmacovigilance research (14). In this study, we employed FAERS-based pharmacovigilance signal detection techniques to systematically evaluate anti-neoplastic agents-related to hepatotoxicity, providing critical evidence-based medical insights with substantial clinical applicability.

## 2 Methods

### 2.1 Data sources

This study mined and analyzed data from the FAERS database (https://www.fda.gov/drugs/drug-approvals-and-databases/fda-adverse-event-reporting-system-faers-database) spanning the first quarter of 2004 to the third quarter of 2024 for mining and analysis. The American Standard Code for Information Interchange (ASCII) data files derived from the FAERS database feature seven subsets: Patient Demographic and Administrative Information (DEMO), Drug Administration Information (DRUG), Report Source Documentation (RPRS), Adverse Event Coding (REAC), Drug Therapy Records (THER), Outcome Data (OUTC) and Drug Indication Records (INDI).

### 2.2 Data processing

**Figure 1:**
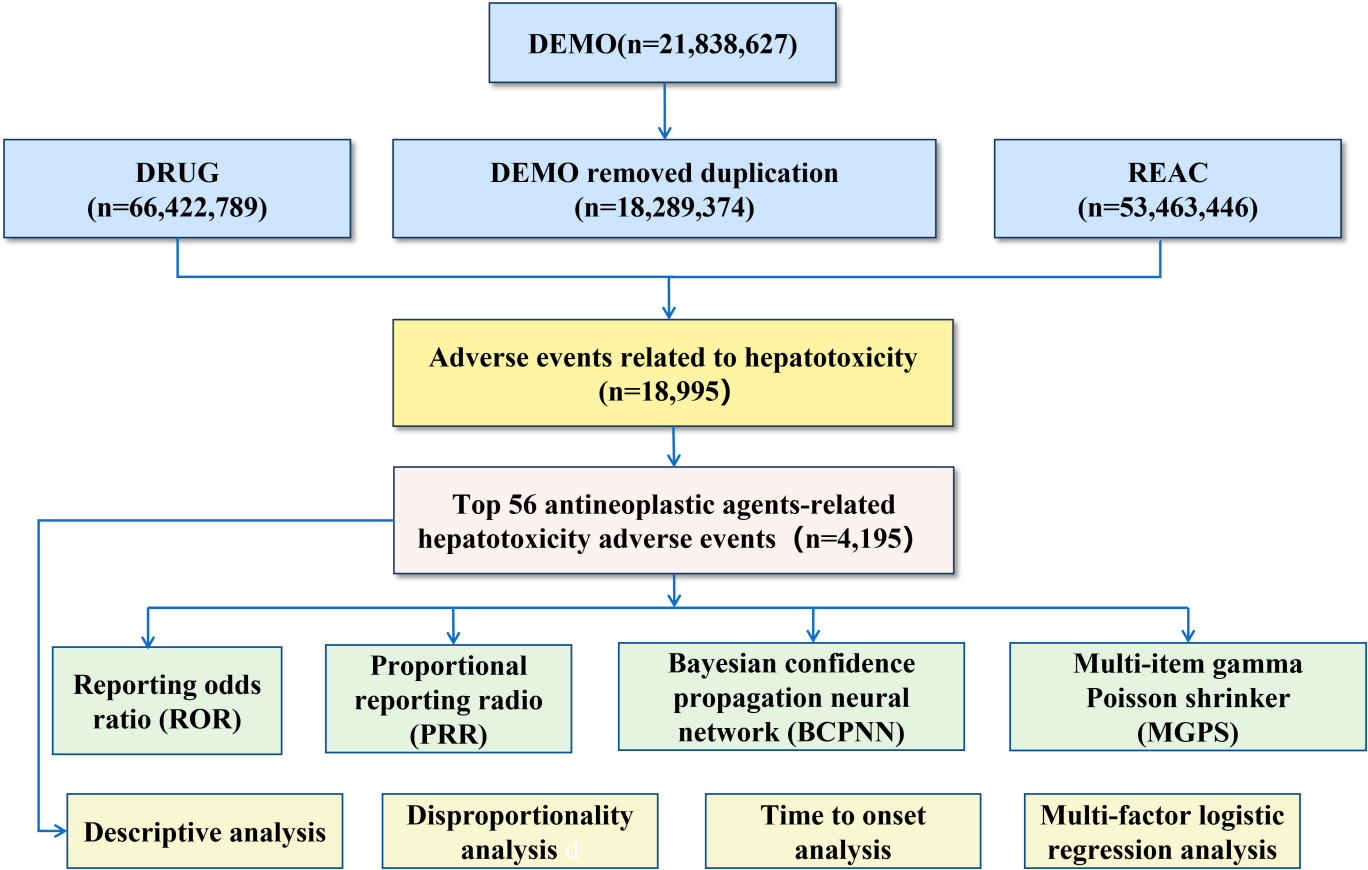
Flow diagram depicting data extraction and cleaning.

Duplicate reports were removed according to FDA de-duplication guidelines, retaining the latest version for cases with identical medical record numbers. Associations between files were established using the PRIMARY ID identifier. Primary Suspect (PS) drugs were identified by searching for the Preferred Term (PT) “hepatotoxicity” standardized via the Medical Dictionary for Regulatory Activities (MedDRA). Drug names were further standardized using the Anatomical Therapeutic Chemical (ATC) system (https://atcddd.fhi.no/atc_ddd_index/). The workflow for extracting and cleansing adverse events associated with hepatotoxicity and anti-neoplastic agents is illustrated in **Figure 1**.

### 2.3 Regression analysis

Univariate analysis was performed on suspected drugs with the inclusion criteria of a 95% confidence interval (CI) lower limit for the reporting odds ratio (ROR) >1, event count >100, and adjusted p-value <0.01. Drugs achieving statistical significance (p < 0.01) in univariate analysis were further subjected to least absolute shrinkage and selection operator (LASSO) regression. A multivariate logistic regression model was constructed using drug variables identified by LASSO combined with baseline patient characteristics as independent variables to identify specific risk factors associated with hepatoxicity and anti-neoplastic agents.

### 2.4 Time to onset analysis

Time-to-onset (TTO) was defined as the interval between the drug initiation date (START_DT in the THER file) and the adverse event occurrence date (EVENT_DT in the DEMO file). Furthermore, inaccurate, missing, or erroneously entered dates were excluded from the analysis. In addition, TTO data were analyzed using the median, interquartile range (IQR), and Weibull shape parameter (WSP). The Weibull distribution is characterized by two parameters: a scale parameter (α) and a shape parameter (β). Three distinct failure patterns were identified based on β values and their 95% CIs: the early failure type: associated with a decreasing adverse drug event (ADE) risk over time, defined as β < 1 (95% CI < 1), the random failure type, representing a constant ADE risk over time, defined as β approaching 1 (95% CI encompassing 1), and the wear-out failure type, indicating an increasing ADE risk over time and defined as β > 1 (95% CI > 1).

### 2.5 Statistical analysis

Adverse event signals were analyzed using four pharmacovigilance algorithms: Reporting Odds Ratio (ROR), Proportional Reporting Ratio (PRR), Bayesian Confidence Propagation Neural Network (BCPNN) and Multi-item Gamma Poisson Shrinker (MGPS). The ROR method served as the primary statistical measure, with higher values indicating stronger associations between anti-neoplastic agents and the risk of hepatotoxicity. Detailed computational formulas for these methods are provided in **Tables 1** and **2**.

**Table 1:** Two-by-two contingency table for disproportionality analyses.

Type of drug	*N* of target adverse events	*N* of other adverse events	Total
Target drug	*a*	*b*	*a+b*
All other drugs	*c*	*d*	*c+d*
Total	*a+c*	*b+d*	*a+b+c+d*

**Table 2:** Overview of the main algorithms used for signal detection.

Algorithm	Publicity	Standard for generating signals
ROR	$ROR=\frac{a/c}{b/d}$	Lower 95% CI>1, *N*≥3
PRR	$PRR=\frac{a/(a+b)}{c/(c+d)}$	χ^2^≥4, PRR≥2, *N*≥3
	$x^{2}=\sum [{\left(O-E\right)}^{2}/E];O=a,E=(a+b)(a+c)/(a+b+c+d)$	
MGPS	$EBGM=\frac{a(a+b+c+d)}{\left(a+b)(a+c\right)}$	Lower 95% CI>2
BCPNN	$IC=\text{l}og_{2}\frac{a(a+b+c+d)}{\left(a+b)(a+c\right)}$	Lower 95% CI>0, *N*>0

## 3 Results

### 3.1 Descriptive analysis

**Figure 2:**
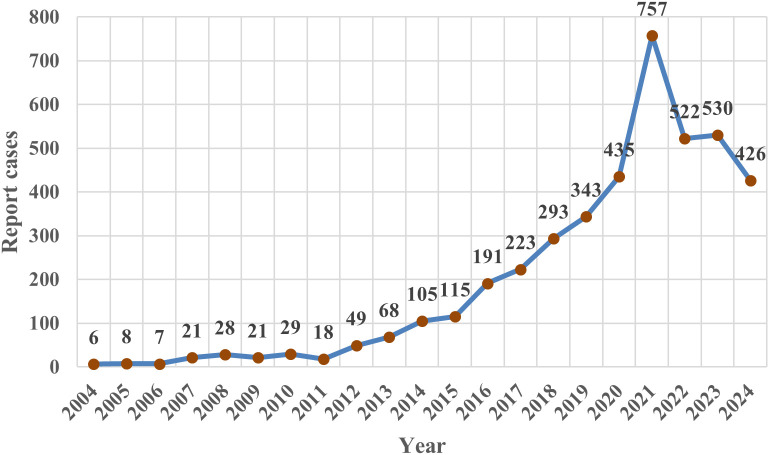
Number of annual reports.

Following data mining, 56 anti-neoplastic agents were found to be positively correlated with hepatotoxicity, as evidenced by 4,195 reports (**Table 3**). Most hepatotoxic adverse events (AEs) related to anti-neoplastic agents originated from the United States (18.30%) and Canada (15.30%). After excluding patients with missing age data (n = 1,716, 40.90%), the median age was 56 years, with the largest proportion of participants (36.00%) concentrated in the 18–64.9 years age group. Body weight was mostly concentrated in the 50–100 kg range (n=558, 13.3%). Following the exclusion of cases with undocumented gender (n = 1,125, 26.80%), female patients (n = 1,949, 46.50%) predominated over males (n = 1,121, 26.70%). A total of 627 patients (14.95%) experienced fatal or life-threatening outcomes. As illustrated in **Figure 2**, the annual number of reported hepatotoxic AEs associated with anti-neoplastic agents exhibited an overall upwards trend from 2004, peaking in 2021 with 757 reported cases.

**Table 3:** Clinical characteristics of reports with hepatotoxicity.

Characteristics	Case number (n)	Case proportion (%)
Gender		
Female	1949	46.46%
Male	1121	26.72%
Missing	1125	26.82%
Age		
<18	237	5.65%
>85	21	0.50%
18∼64.9	1512	36.04%
65∼85	709	16.90%
Missing	1716	40.91%
Weight		
<50 kg	97	2.31%
>100 kg	64	1.53%
50∼100 kg	558	13.30%
Missing	3476	82.86%
Reporting region		
United States	766	18.26%
Canada	568	13.54%
Italy	348	8.30%
Spain	316	7.53%
Germany	252	6.01%
Outcomes		
Death	439	10.46%
Disability	21	0.50%
Hospitalization	725	17.28%
Life-Threatening	188	4.48%
Other and Unknown	2822	67.27%
Totality	4195	100.00%

### 3.2 Disproportionality analysis

**Figure 3:**
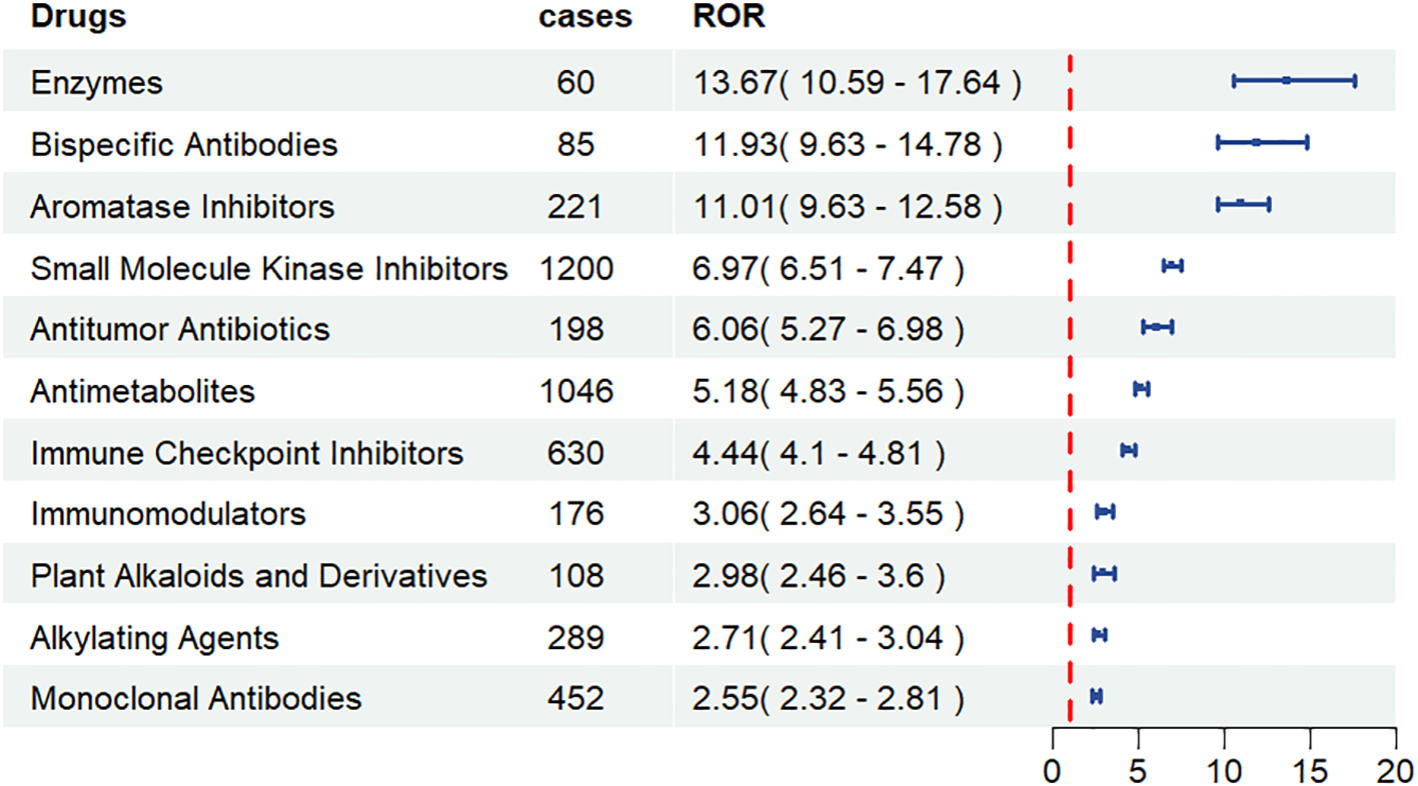
Forest plot of ROR values for different type of anti-neoplastic agents associated with hepatotoxicity in the FAERS database.

Following data cleaning and analysis, a total of 56 anti-neoplastic agents were identified with positive hepatotoxicity signals. The top 30 anti-neoplastic agents ranked by ROR values are presented in **Table 4**. The five highest-ranking agents were: mercaptopurine (ROR = 26.57, 95% CI: 20.84–33.89), pegaspargase (ROR = 13.67, 95% CI: 10.59–17.64), blinatumomab (ROR = 11.93, 95% CI: 9.63–14.78), letrozole (ROR = 11.01, 95% CI: 9.63–12.58) and dasatinib (ROR = 10.25, 95% CI: 9.14–11.50). Subsequent classification of the top 30 anti-neoplastic agents revealed that small molecule kinase inhibitors accounted for the highest number of hepatotoxicity cases and enzymes had the highest ROR values (**Figure 3**). Furthermore, among these 56 anti-neoplastic agents, the package inserts of 45 drugs contain warnings regarding hepatotoxicity risks, while the remaining 11 drug’s package inserts do not include hepatotoxicity risk alerts. These eleven drugs without hepatotoxicity warnings are: letrozole, doxorubicin, carboplatin, trastuzumab, blinatumomab, cytarabine, dabrafenib, pemetrexed, melphalan, vismodegib and midostaurin.

**Table 4:** The top 30 anti-neoplastic agents associated with ADE of hepatotoxicity.

Ranking	ATC code	Drug	Cases	ROR (95% CI)	PRR (χ;^2^)	EBGM (EBGM05)	IC (IC025)
1	L01BB02	Mercaptopurine	67	26.57 (20.84-33.89)	25.89 (1599.01)	25.8 (21.05)	4.69 (4.33)
2	L01XX24	Pegaspargase	60	13.67 (10.59-17.64)	13.49 (692.46)	13.45 (10.87)	3.75 (3.38)
3	L01FX07	Blinatumomab	85	11.93 (9.63-14.78)	11.8 (837.18)	11.75 (9.82)	3.55 (3.24)
4	L02BG04	Letrozole	221	11.01 (9.63-12.58)	10.9 (1965.08)	10.78 (9.64)	3.43 (3.23)
5	L01EA02	Dasatinib	299	10.25 (9.14-11.5)	10.16 (2431.52)	10.01 (9.09)	3.32 (3.15)
6	L01EF02	Ribociclib	141	8.42 (7.13-9.94)	8.36 (907.11)	8.3 (7.22)	3.05 (2.81)
7	L01EF03	Abemaciclib	86	7.3 (5.9-9.03)	7.25 (461.7)	7.22 (6.04)	2.85 (2.54)
8	L01EX03	Pazopanib	174	7.05 (6.07-8.19)	7.01 (888.74)	6.95 (6.13)	2.8 (2.58)
9	L01EC01	Vemurafenib	65	6.87 (5.38-8.77)	6.82 (322.34)	6.8 (5.55)	2.77 (2.41)
10	L01BC01	Cytarabine	74	6.13 (4.88-7.71)	6.1 (314.5)	6.08 (5.02)	2.6 (2.27)
11	L01DB01	Doxorubicin	198	6.06 (5.27-6.98)	6.03 (823.4)	5.98 (5.32)	2.58 (2.37)
12	L01BA01	Methotrexate	766	6.01 (5.59-6.46)	5.98 (3052.02)	5.78 (5.44)	2.53 (2.42)
13	L01EC02	Dabrafenib	72	5.49 (4.35-6.92)	5.46 (261.75)	5.45 (4.48)	2.45 (2.11)
14	L01FF02	Pembrolizumab	245	5.23 (4.61-5.93)	5.2 (822.33)	5.15 (4.63)	2.36 (2.18)
15	L01FX04	Ipilimumab	91	5.15 (4.19-6.34)	5.13 (301.66)	5.11 (4.3)	2.35 (2.05)
16	L01EH01	Lapatinib	62	5.04 (3.92-6.47)	5.01 (198.84)	5 (4.06)	2.32 (1.96)
17	L01FF05	Atezolizumab	106	4.87 (4.02-5.9)	4.85 (322.47)	4.83 (4.11)	2.27 (1.99)
18	L01BA04	Pemetrexed	61	4.81 (3.74-6.19)	4.79 (182.52)	4.78 (3.87)	2.26 (1.89)
19	L01FD01	Trastuzumab	146	4.31 (3.66-5.08)	4.3 (367.19)	4.27 (3.73)	2.1 (1.86)
20	L01EK01	Axitinib	54	3.53 (2.7-4.62)	3.52 (97.39)	3.52 (2.81)	1.81 (1.42)
21	L01XA02	Carboplatin	147	3.39 (2.88-3.99)	3.38 (245.11)	3.36 (2.94)	1.75 (1.51)
22	L01EX09	Nintedanib	71	3.35 (2.66-4.24)	3.35 (116.47)	3.34 (2.75)	1.74 (1.4)
23	L01AA01	Cyclophosphamide	97	3.2 (2.62-3.91)	3.19 (145.54)	3.18 (2.69)	1.67 (1.38)
24	L01FA01	Rituximab	306	3.15 (2.82-3.53)	3.15 (441.13)	3.11 (2.83)	1.64 (1.47)
25	L01EA01	Imatinib	176	3.06 (2.64-3.55)	3.05 (240.84)	3.03 (2.68)	1.6 (1.38)
26	L04AC07	Tocilizumab	176	3.06 (2.64-3.55)	3.06 (241.4)	3.04 (2.68)	1.6 (1.38)
27	L01CD01	Paclitaxel	108	2.98 (2.46-3.6)	2.97 (140.47)	2.96 (2.53)	1.57 (1.29)
28	L01BC05	Gemcitabine	78	2.92 (2.34-3.65)	2.92 (97.95)	2.91 (2.41)	1.54 (1.21)
29	L01FF01	Nivolumab	188	2.89 (2.5-3.33)	2.88 (228.65)	2.86 (2.54)	1.52 (1.31)
30	L01XA03	Oxaliplatin	88	2.76 (2.24-3.4)	2.75 (98.03)	2.75 (2.3)	1.46 (1.15)

ADE, adverse drug event; ATC, Anatomical Therapeutic Chemical; CI, confidence interval; ROR, Reporting Odds Ratio; PRR, Proportional Reporting Ratio; BCPNN, Bayesian Confidence Propagation Neural Network; MGPS, Multi-item Gamma Poisson Shrinker.

### 3.3 Risk factors for hepatotoxicity related to anti-neoplastic agents

**Figure 4:**
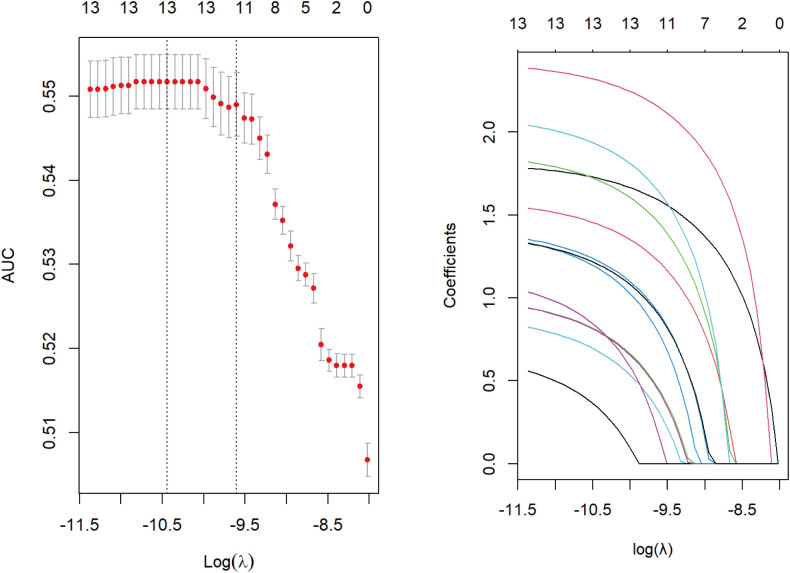
Results of LASSO regression analysis. LASSO, least absolute shrinkage and selection operator.

**Figure 5:**
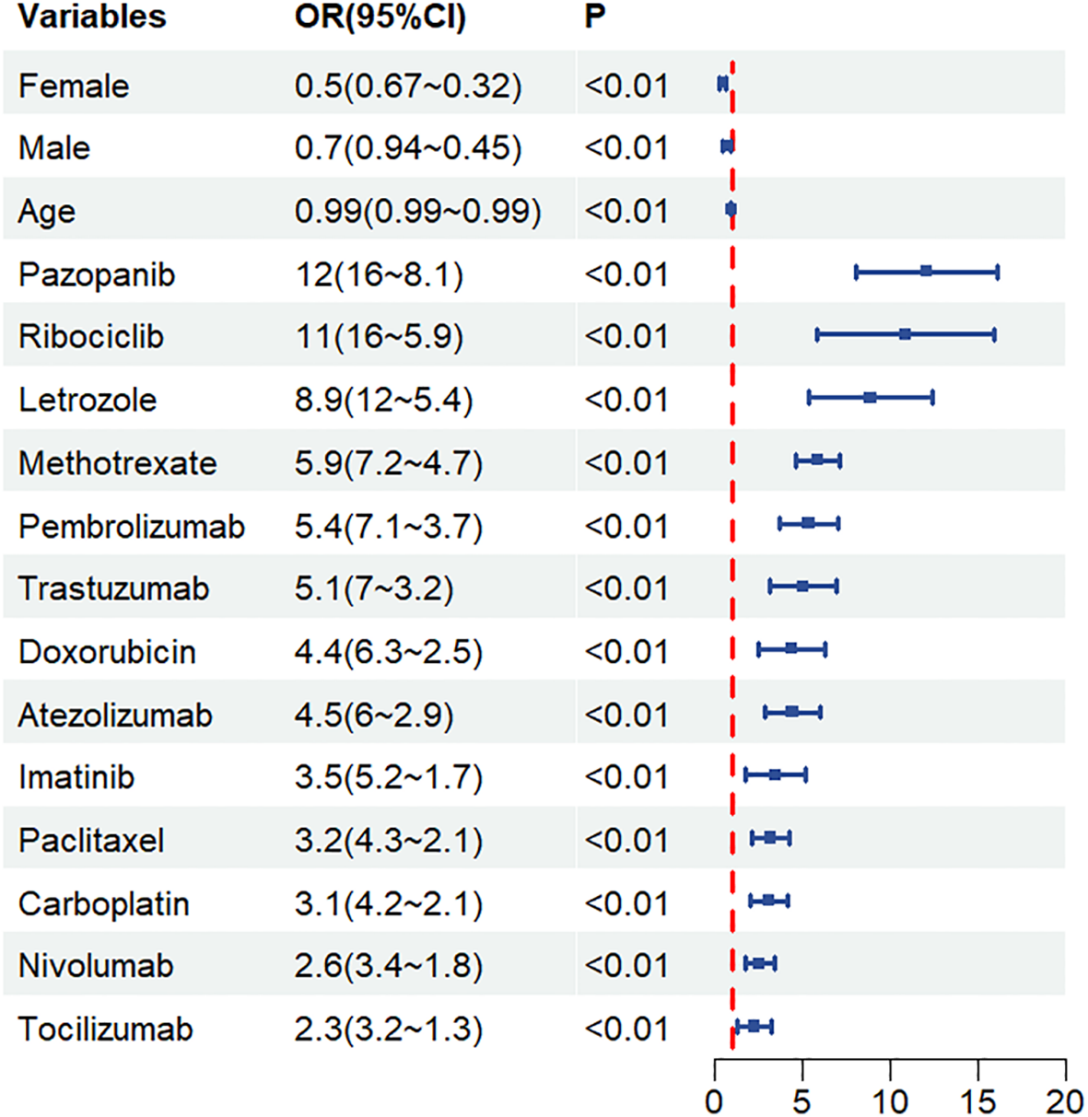
Results arising from multi-factor logistic regression analysis. CI, confidence interval; OR, odds ratio; P-adjust, p-value after Bonferroni correction; P-adjust<0.01, statistically significant.

Suspected drugs with >100 case reports, a lower 95% CI limit of the ROR >1 and p-adjust < 0.01 were extracted for univariate analysis. Drugs with p < 0.01 in univariate analysis were subjected to LASSO regression, and a total of 13 drugs were identified (**Figure 4**). Multi-factor logistic regression analysis of these drugs was performed in combination with patient information (**Figure 5**). Analysis showed that 13 drugs, including pazopanib, ribociclib, letrozole, methotrexate, pembrolizumab, trastuzumab, doxorubicin, atezolizumab, imatinib, paclitaxel, carboplatin, nivolumab and tocilizumab were identified as independent risk factors for anti-neoplastic agents associated with hepatotoxicity.

### 3.4 Time to onset analysis

**Figure 6:**
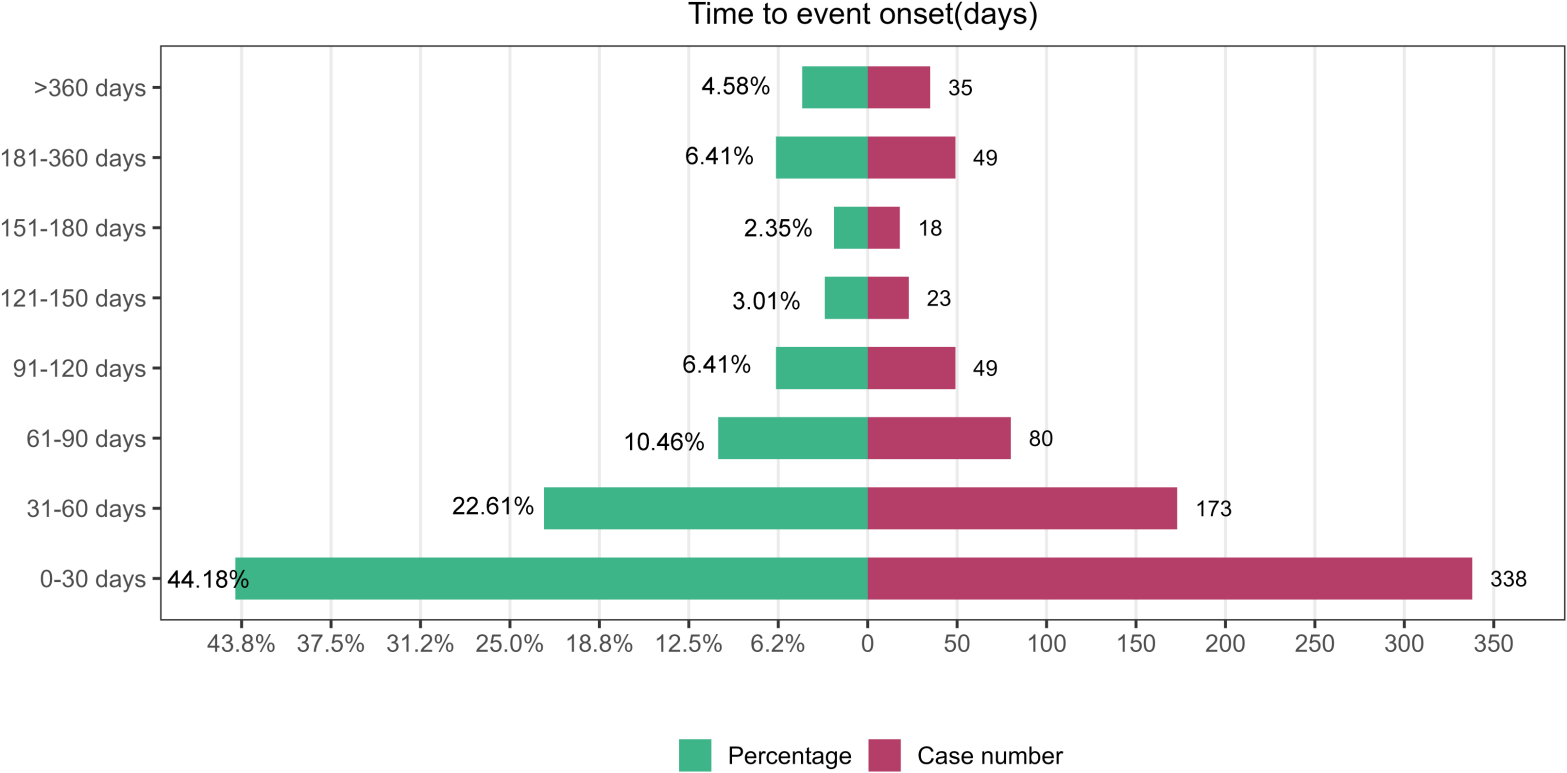
Time-to-onset (TTO) of anti-neoplastic-associated hepatotoxicity.

**Figure 7:**
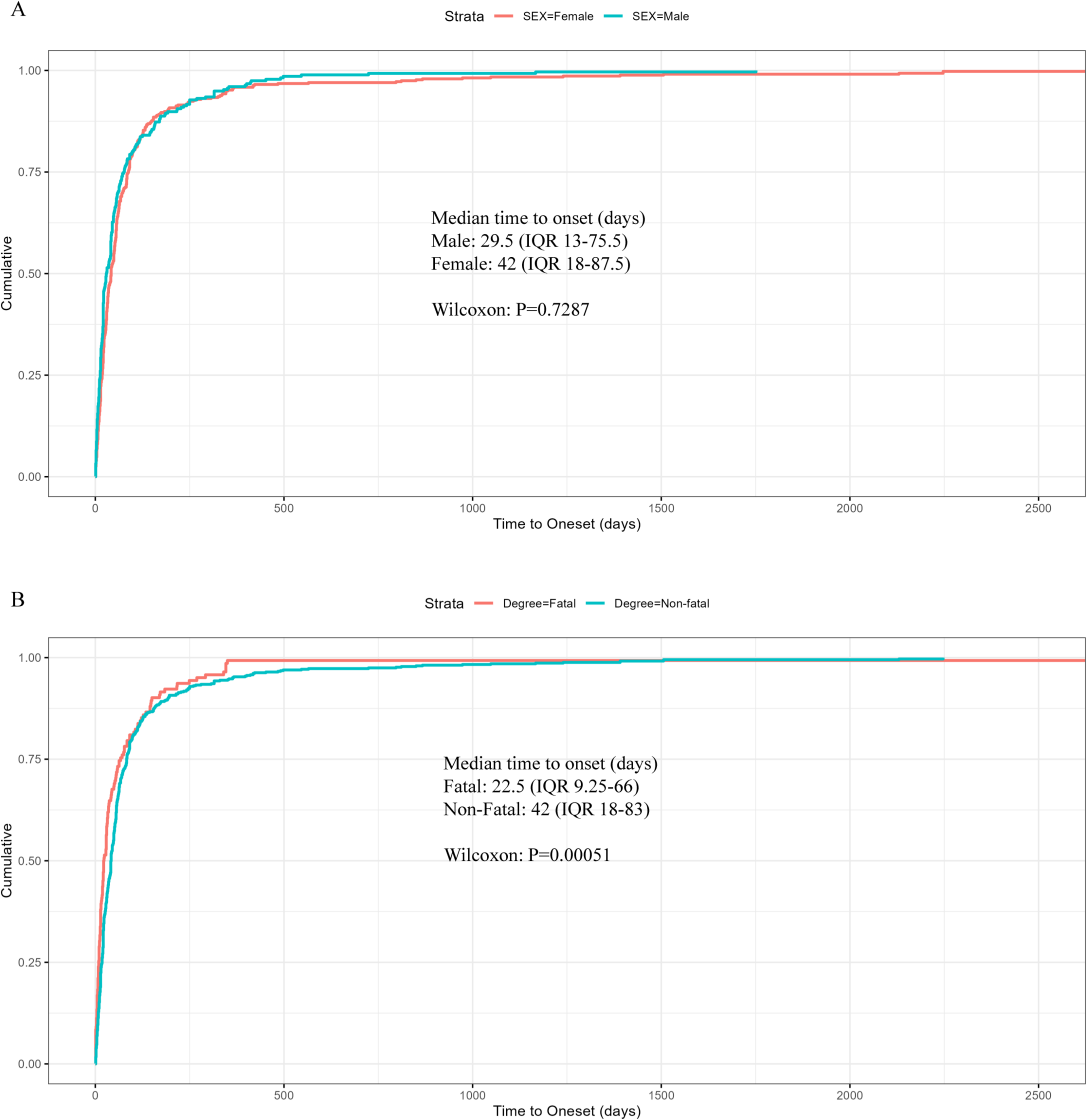
Cumulative distribution curves demonstrating the time-to-onset (TTO) for anti-neoplastic-related hepatotoxicity among different subgroups. **(A)**Sex. **(B)** Fatal status. Statistical significance was assessed using the non-parametric Wilcoxon rank sum test.

To enhance the credibility of our TTO assessment, data inconsistencies and missing values were systematically removed, yielding a reduced cohort for subsequent analysis compared to the original dataset. **Figure 6** illustrates the temporal distribution of events, with 44.18% of subjects (n=338) experiencing onset within the first 30 days following the initiation of treatment. **Table 5** presents the finding of TTO and WSP analysis for the top 30 anti-neoplastic agents associated with hepatotoxicity. According to Weibull assessment, most of the top 30 drugs were random failure models, indicating that hepatotoxicity could occur at any point during treatment without specific time dependency. Cumulative distribution curves demonstrated the onset time for anti-neoplastic agents associated with hepatotoxicity among the different subgroups (**Figure 7**). The median onset time for hepatotoxicity associated with male gender was 29.5 days (IQR:13–75.5), compared to 42 days (IQR: 18–87.5) for female cases; there was no significant difference between males and females (*P* = 0.7287). Significant differences were detected for the TTO of hepatotoxicity in terms of fatal status. The median onset time was 22.5 days (IQR 9.25–66) for fatal cases; this was significantly different than the 42 days (IQR:18–83) for non-fatal cases (*P* = 0.00051).

**Table 5:** TTO for the top 30 anti-neoplastic associated with ADE of hepatotoxicity.

SN	Drug	Cases	TTO (days)		Weibull distribution				Failure type
					Scale parameter		Shape parameter		
		n	Median	IQR	α	95%CI	β	95%CI	
1	Mercaptopurine	67	72	10.5-315	235.14	17.16- 453.11	0.43	0.31-0.55	Early Failure
2	Pegaspargase	60	1	1-7.5	4.66	-2.33-11.64	0.80	0.11-1.50	Random Failure
3	Blinatumomab	85	4	1-10	11.30	-0.87-23.46	0.54	0.34- 0.74	Early Failure
4	Letrozole	221	51	27.5-91	86.35	64.58-108.12	0.93	0.78-1.07	Random Failure
5	Dasatinib	299	64	36-94.5	68.87	-1.38-139.12	1.16	0.03- 2.29	Random Failure
6	Ribociclib	141	26	10.5-114.5	70.14	-9.90-150.19	0.69	0.30- 1.08	Random Failure
7	Abemaciclib	86	60	21-84	76.99	16.45-137.53	0.88	0.45-1.31	Random Failure
8	Pazopanib	174	40.5	21-49	49.52	30.08-68.97	1.13	0.81-1.44	Random Failure
9	Vemurafenib	65	43.5	12-56.25	48.53	19.13-77.92	1.08	0.57- 1.59	Random Failure
10	Cytarabine	74	15	12-15	15.77	10.28-21.26	2.64	0.77-4.52	Random Failure
11	Doxorubicin	198	1	–	–	–	–	–	–
12	Methotrexate	766	72	10.5-315	235.14	17.16-453.11	0.43	0.31- 0.55	Early Failure
13	Dabrafenib	72	107	58-195	184.32	83.89-284.74	0.93	0.61-1.24	Random Failure
14	Pembrolizumab	245	50.5	21-71.75	73.55	36.59-110.50	0.84	0.61-1.08	Random Failure
15	Ipilimumab	91	44	22-96	82.92	41.31-124.52	0.91	0.63-1.18	Random Failure
16	Lapatinib	62	42	36-91	77.21	48.73-105.70	1.46	0.91-2.01	Random Failure
17	Atezolizumab	106	27	19.5-112.5	74.98	37.45-112.50	0.75	0.55-0.94	Early Failure
18	Pemetrexed	61	41	41-41	45.17	37.09-53.25	3.10	1.94-4.26	Wear-out Failure
19	Trastuzumab	146	49	23-101	212.76	32.21-393.31	0.54	0.37-0.71	Early Failure
20	Axitinib	54	19	11.5-37	27.13	-1.67-55.93	1.12	0.09-2.16	Random Failure
21	Carboplatin	147	45	31-78.5	63.91	40.60-87.23	1.17	0.78-1.56	Random Failure
22	Nintedanib	71	9.5	3.5-71	33.34	-13.08-79.77	0.61	0.23-0.99	Early Failure
23	Cyclophosphamide	97	37	16-63	39.10	-8.55-86.75	0.84	0.12-1.55	Random Failure
24	Rituximab	306	25	9-39	48.73	-13.87-111.34	0.61	0.28-0.94	Early Failure
25	Imatinib	176	75.5	53.75-87.75	73.85	37.88-109.82	2.09	0.23-3.95	Random Failure
26	Tocilizumab	176	18	7.25-40.75	29.90	-1.40-61.20	0.99	0.24-1.75	Random Failure
27	Paclitaxel	108	56	32-73	55.94	42.16-69.72	1.82	1.18-2.46	Wear-out Failure
28	Gemcitabine	78	19.5	5.5-54.5	35.06	-11.22-81.33	0.79	0.19-1.39	Random Failure
29	Nivolumab	188	47.5	20.25-71	68.38	50.14-86.61	1.06	0.85-1.27	Random Failure
30	Oxaliplatin	88	10.5	4-14	16.36	3.01-29.71	0.90	0.46-1.35	Random Failure

SN, Serial Number; TTO, Time-to-onset; ADE, adverse drug event; CI, confidence interval; IQR, interquartile range.

## 4 Discussion

Hepatotoxicity represents a well-documented adverse reaction associated with anti-neoplastic therapies. However, there is a scarcity of large-scale studies investigating the potential correlation between anti-neoplastic agents and hepatoxicity. In this study, we mined data from the FAERS database to identify anti-neoplastic agents associated with hepatotoxicity. Small molecule kinase inhibitors accounted for the highest proportion of case reports, with submissions predominantly originating from Europe and North America. Demographic analysis demonstrated that most AEs involved females, and that most AEs were concentrated in patients aged 18–64.9 years, with a median age of 56 years. Temporally, 44.18% of events occurred within one month of the initiation of treatment. Notably, life-threatening outcomes or fatalities were documented in 14.95% of cases.

In the present study, we revealed a higher incidence of hepatotoxicity associated with anti-neoplastic agents in female patients when compared to males. This disparity may have arisen because of sex-specific physiological variations, including differences in body weight, fat distribution, hepatic function and gastrointestinal physiology (15). When administering equivalent drug doses, these factors may impair metabolic efficiency in females, thereby elevating the risk of adverse drug reactions. Furthermore, hepatotoxic AEs associated with anti-neoplastic agents predominantly occurred in individuals aged 18–64.9 year. Previous research showed that advanced age represents a critical risk factor for DILI (16). Elderly patients are predisposed to hepatotoxicity due to age-related immune decline, higher burdens of comorbidity and diminished drug metabolism capacity (17). However, two prospective studies from the United States and Spain failed to identify significant differences in the incidence of hepatotoxicity when compared between elderly and adolescent populations, although elderly individuals exhibited a higher incidence of cholestatic liver injury than younger adults (18). These findings might be related to different geographical environments.

With regards to the TTO for hepatotoxicity, we found that over half of all hepatotoxicity cases occurred within the first two months of treatment, thus indicating that most hepatotoxicity events arise early, consistent with previous studies (19). Moreover, the median TTO was shorter in fatal cases than in non-fatal cases, thus indicating more rapid disease progression in fatal outcomes. This disparity may be attributed to the critical nature of these cases compounded by poor baseline health status, particularly pre-existing liver damage, which could enhance susceptibility to drug-induced AEs and thereby accelerate pathological deterioration. Collectively, these findings highlight the importance of early identification and therapeutic intervention for drug-induced hepatotoxicity to mitigate disease escalation. The WSP test further revealed that the occurrence of hepatotoxicity for most drugs was not time-dependent, thus suggesting that hepatotoxicity may arise at any phase during the treatment process. Therefore, although enhanced monitoring of hepatic function is critical during the initial phase of chemotherapy, continuous surveillance throughout the entire course of treatment is essential to ensure that no potential cases are missed.

Further classification revealed that enzymes had the highest ROR values. Pegaspargase, a covalent conjugate of polyethylene glycol (PEG) and L-asparaginase, serves as a first-line agent for the treatment of acute lymphoblastic leukemia (ALL). However, hepatotoxicity frequently occurs during pegaspargase therapy. A previous clinical study demonstrated that asparaginase may induce liver injury accompanied by jaundice, typically characterized by short latency, marked steatosis, and prolonged cholestasis, potentially attributable to the inhibition of hepatic protein synthesis secondary to the depletion of asparagine (20). Interestingly, a retrospective analysis of 141 pegaspargase-treated patients revealed that administration on day 15 of the ALL induction phase significantly reduced the risk of high-grade hepatotoxicity when compared to dosing on day 4, while age and elevated BMI were further identified as independent risk factors for severe hepatotoxicity (21, 22). Furthermore, a pharmacokinetic study of pegaspargase demonstrated that an interval of more than four weeks between successive administrations is required to prevent drug accumulation in adults (23). Therefore, delayed administration, BMI optimization and controlled dosing intervals should be considered in clinical practice to mitigate pegaspargase-associated adverse effects.

Multi-factor analysis further showed that small molecule kinase inhibitors (pazopanib, ribociclib and imatinib), immune checkpoint inhibitors (pembrolizumab, atezolizumab and nivolumab), letrozole, methotrexate, doxorubicin, paclitaxel, tocilizumab were all risk factors for the induction of drug-related hepatotoxicity.

In this study, we revealed that small molecule kinase inhibitors accounted for the highest proportion of drug-induced hepatotoxicity. Pazopanib, as a tyrosine kinase inhibitor (TKI), is primarily utilized to treat patients with advanced renal cell carcinoma. While TKIs represent breakthrough therapies for several malignancies, these drugs are also associated with a high incidence of hepatotoxicity. As of April 2021, six approved TKIs (11%) carried black box warnings for hepatotoxicity, and an additional 25 agents (46%) included hepatotoxicity alerts in their prescribing information (24). A case report highlighted the fact that two patients developed severe hepatotoxicity within six weeks of the initiation of pazopanib and that one of these patients succumbed to this toxicity (25). In terms of mechanistic action, a previous study of sunitinib-induced hepatotoxicity demonstrated that sunitinib caused regional damage to hepatocytes, bile duct cells, and hepatic sinusoidal endothelial cells in the portal vein area; this process was associated with hallmark cellular events including autophagy, apoptosis, and mitochondrial injury (26). Beyond such intrinsic drug toxicity, hepatotoxicity can also be exacerbated by drug-drug interactions. For instance, the co-administration of erlotinib, which is primarily metabolized by CYP3A4, with strong inhibitors of this enzyme (e.g., ketoconazole or ritonavir) has been shown to elevate its systemic exposure, thereby increasing the risk of DILI (27). These findings highlight the necessity for rigorous monitoring of liver function parameters during the clinical application of such small molecule kinase inhibitors.

The advent of ICIs represented a groundbreaking advancement in oncology and was recognized by the 2018 Nobel Prize in Physiology or Medicine. ICIs are monoclonal antibodies that target immune checkpoint molecules, thus providing an immunotherapeutic approach for numerous advanced malignancies. The U.S. FDA approved the first ICI, ipilimumab, in 2011 for the treatment of metastatic melanoma. During ICI therapy, 2–25% of patients may develop hepatic dysfunction characterized by abnormal hepatocellular serum biochemical parameters (28). Immune-related adverse events (irAEs) limit the clinical application of ICIs, with hepatotoxicity constituting a critical form of irAE. FDA clinical trials and observational studies have shown that up to 16% of patients receiving ICIs may experience immune-mediated liver injury, although the incidence of this condition varies substantially depending on ICI class, dosage and therapeutic regimens (29). When evaluating hepatotoxicity associated with ICIs, it is crucial to distinguish direct drug-induced liver injury from potentially confounding irAEs. Immune-mediated pancreatitis may lead to secondary cholestasis due to biliary obstruction (30), whereas drug-induced autoimmune hepatitis (DIAIH) presents with clinical features that overlap between classic drug-induced liver injury and autoimmune hepatitis. A reliable diagnosis of DIAIH requires the concurrent use of both the RUCAM and the simplified AIH score (31). Misclassification of these irAEs could compromise the specificity of hepatotoxicity signals attributed directly to ICIs. The mechanisms underlying checkpoint inhibitor-induced immune-mediated hepatotoxicity have yet to be fully elucidated. Notably, Johncilla et al. (32) reported the increased expression of T-cell activation markers in liver specimens acquired from 11 patients who developed hepatic injury following ipilimumab treatment, thus suggesting that this may represent one potential mechanism underlying ICI-induced hepatotoxicity. Furthermore, analysis of a mouse model demonstrated that ICI treatment induced liver damage as well as hepatocyte apoptosis and activation of the Nod-like receptor protein 3 (NLRP3) inflammasome (33).

Letrozole, a hormonal anti-neoplastic agent, is a highly selective aromatase inhibitor and serves as a first-line treatment for locally advanced or postmenopausal breast cancer. However, cases of letrozole-associated hepatotoxicity have been reported. For example, existing literature documents the case of a 70-year-old female patient who developed jaundice and markedly elevated levels of hepatic transaminases after three months of letrozole treatment, with liver biopsy confirming drug-induced hepatotoxicity. Her liver function gradually returned to normal within three weeks of drug discontinuation (34). A phase II clinical trial further reported that letrozole may increase the risk of hepatotoxicity (35). Although reports of letrozole-induced liver injury remain relatively rare, the close monitoring of liver function is strongly recommended during the clinical administration of letrozole.

Methotrexate, an anti-metabolite and anti-neoplastic agent, is primarily used for maintenance therapy in patients with ALL. Hepatotoxicity has been established as a severe adverse reaction associated with methotrexate. A meta-analysis of 32 studies, involving 13,177 patients, identified a significant association between the use of methotrexate and an increased risk of hepatic AEs (12). Furthermore, a randomized controlled trial (RCT) revealed significant elevations in the levels of liver enzymes in methotrexate-treated patients at 6- and 12-months intervals (13). Consistent with previous evidence, our analysis identified methotrexate as a risk factor for drug-induced liver injury. Furthermore, this hepatotoxicity was substantiated by pharmacogenomic findings linking polymorphisms in genes such as *ABCC2*, *MTHFR*, and *SXR* and *MTX*-induced toxicities, including hepatotoxicity and myelosuppression, in pediatric solid tumors (36). Mechanistically, *in vitro* studies have demonstrated that ferroptosis and oxidative stress contribute directly to MTX-induced liver injury (37).

The FAERS database comprises spontaneous reports of suspected adverse reactions that typically lack rigorous diagnostic validation for DILI. This represents a major methodological limitation, as the absence of a standardized causality assessment tool, such as the updated RUCAM, which is specifically validated for DILI, hinders the ability to distinguish true, idiosyncratic DILI from other causes of liver test abnormalities, including confounding immune-related adverse events or underlying diseases (31, 38). Consequently, hepatotoxicity signals derived from such analyses remain associative rather than causally verified, limiting their mechanistic interpretation and clinical applicability. To enhance the quality and specificity of DILI data in pharmacovigilance, we strongly recommend that future case reporting and analysis mandate the application of the updated RUCAM. Inclusion of cases in registries or signal-detection studies should be contingent upon causality assessment using this validated instrument. Widespread adoption of RUCAM would improve the scientific rigor of pharmacovigilance research and yield more reliable data for characterizing the clinical and phenotypic spectrum of anti-neoplastic agent-induced DILI.

### 4.1 Limitations

This study has several limitations that should be considered. First, a key limitation of our study is the inability to assess causality using the RUCAM scale due to insufficient clinical detail in FAERS reports. This highlights the need for future clinical studies to confirm our pharmacovigilance signals with established diagnostic tools. Second, AE reports in the FAERS database were collected by spontaneous reporting systems, which may have led to under-reporting, reporting biases, and frequent omission of critical patient demographic information—particularly regarding age, sex, weight, and underlying medical conditions—due to variations in pharmacovigilance practices across different regions and countries. Third, the disproportionality analysis method employed in this research can only identify statistical associations by quantitative signals. Such findings do not necessarily imply a definitive clinical causal relationship between the reported AEs and anti-neoplastic agents. Furthermore, some novel anti-neoplastic agents have been on the global market for a relatively short period of time, thus resulting in a relatively incomplete set of safety surveillance data. Therefore, further clinical studies are now warranted to validate these findings through more rigorous pharmacoepidemiological investigations.

## 5 Conclusion

This study represents the first pharmacovigilance investigation utilizing the FAERS database to systematically analyze hepatotoxicity associated with anti-neoplastic agents. A key finding of our analysis was the contrast between the 56 anti-neoplastic agents significantly associated with hepatotoxicity and the complete lack of hepatotoxicity warnings in the package inserts for eleven of these agents. The median onset of hepatotoxicity in the Fatal group (22.5 days) was shorter than that in the non-fatal group (42 days). Furthermore, WSP analysis showed that 20 of the top 30 drugs by ROR value followed a random failure model, thus suggesting that the onset of hepatotoxicity could happen at any point during treatment, at random. In conclusion, our research provides crucial evidence for the clinical management and prevention of anti-neoplastic agents-induced hepatotoxicity. Nonetheless, given the inherent limitations of spontaneous reporting systems and signal detection methodologies, these findings warrant further validation through longitudinal pharmacoepidemiological studies incorporating comprehensive causality assessments.

## Data Availability

The original contributions presented in the study are included in the article/supplementary material. Further inquiries can be directed to the corresponding author.
